# A Systematic Review and Meta-Analysis of the Effects of Various Physical Activity Interventions in Pregnant Women with Overweight or Obesity

**DOI:** 10.3390/healthcare13243319

**Published:** 2025-12-18

**Authors:** Mingmao Li, Hongli Yu, Guoping Qian, Anna Szumilewicz, Zbigniew Ossowski

**Affiliations:** 1 Faculty of Physical Culture, Gdansk University of Physical Education and Sport, 80-336 Gdansk, Poland; guoping.qian@awf.gda.pl (G.Q.); anna.szumilewicz@awf.gda.pl (A.S.); zbigniew.ossowski@awf.gda.pl (Z.O.); 2 College of Physical Education, Sichuan University of Science & Engineering, Zigong 643000, China

**Keywords:** pregnancy, gestational weight gain, physical activity, overweight and obesity in pregnancy

## Abstract

**Background**: Obesity during pregnancy increases the risk of adverse maternal and neonatal outcomes, and excessive gestational weight gain (GWG) remains highly prevalent worldwide. Although physical activity (PA) interventions have shown potential benefits, evidence on the optimal type, intensity, and duration of exercise for overweight or obese pregnant women remains limited. **Methods**: Electronic searches of EBSCOhost, Embase, PubMed and Web of Science were performed through August 2025 to identify randomized controlled trials comparing PA interventions versus usual prenatal care in overweight or obese pregnant women. Two reviewers independently screened studies, extracted data, and assessed risk of bias using Cochrane ROB domains. Continuous outcomes were pooled using inverse-variance meta-analytic methods and heterogeneity was quantified by I^2^. **Results**: Ten randomized trials (twelve intervention arms) comprising 1150 participants met the inclusion criteria. In the domain of blinding of participants and personnel, three studies (30%) were judged as low risk, while seven (70%) were unclear. PA interventions varied in modality (aerobic, resistance, endurance, walking), setting (clinic, community, home/mHealth), and the intervention period ranges from 10 to 34 weeks. Most interventions (80%) employed moderate intensity, and 30% combined aerobic and resistance training. Results of the meta-analysis showed that the pooled mean GWG was 9.93 ± 5.48 kg in the treatment group and 10.65 ± 5.70 kg in the control group. Overall, PA interventions produced a modest but statistically significant reduction in GWG compared with controls, with negligible between-study heterogeneity (I^2^ = 0%). **Conclusions**: Tailored, moderate-intensity PA may have the potential to modestly reduce GWG. Although 30% included trials employed combined aerobic and resistance training, current evidence is insufficient to establish whether combined modalities are more effective than aerobic-only or resistance-only interventions. However, the current evidence is limited by small trial sizes, methodological variability and geographic concentration in higher-income settings. Larger, rigorously designed RCTs, including evaluations of digital delivery platforms and carefully supervised higher-intensity protocols, are needed to refine exercise prescriptions and inform clinical guidelines.

## 1. Introduction

Obesity has become a major global public health concern. Empirical evidence suggests that obesity during pregnancy markedly elevates the risk of unfavorable health outcomes [1]. Women who are overweight during gestation are more susceptible to complications such as gestational diabetes, hypertensive disorders, preeclampsia, and a greater probability of cesarean section [2]. Furthermore, their offspring are at increased risk of macrosomia, which may predispose them to health challenges both in infancy and throughout later stages of development [3]. In recent years, there has been a notable rise in the prevalence of excessive GWG among pregnant women worldwide. Prevalence rates of excessive gestational weight gain have been documented as 30% in China, 20% in urban India, 50.7% in Australia, and 30% in Brazil [4,5,6]. In Europe, recent large-scale surveillance indicates that the prevalence of excessive GWG ranges between 47% and 51%, reflecting substantial public health burden comparable to other high-income regions [7,8]. These increases are attributed to behavioral and societal determinants, including physical inactivity, dietary modifications, socioeconomic disparities, limited healthcare access, and prevailing cultural norms [9,10].

Evidence from quantitative research demonstrates that interventions targeting lifestyle modification, dietary regulation, and PA are effective in reducing the incidence of pregnancy complications associated with excessive GWG [11,12,13,14]. The PA intervention in particular showed the common methods used in the overweight or obesity pregnant group. The American College of Sports Medicine (ACSM) recommends that pregnant women engage in a minimum of 150 min of moderate-intensity aerobic activity per week, such as brisk walking or swimming [15]. Incorporating moderate resistance training has been shown to enhance muscular strength, support postural stability, alleviate pregnancy-related low back pain, and decrease the likelihood of developing metabolic syndrome during pregnancy. In line with the guidance of both the ACSM and the World Health Organization (WHO), pregnant women are encouraged to participate in diverse forms of physical activity, including aerobic exercise, resistance training, flexibility exercises, as well as daily functional movements [15,16]. Empirical evidence further indicates that regular engagement in moderate-intensity aerobic activity can improve cardiorespiratory fitness, mitigate the risks of gestational diabetes and hypertensive disorders, and assist in regulating GWG [17]. Some studies have shown that pregnant women who combine resistance training have better results in controlling GWG and lowering cesarean section rates than aerobic exercise alone [18]. Flexibility training increases joint mobility, reduces muscle tension and improves comfort for pregnant women [19,20]. Exercise regimen needs to be individualized due to large individual differences. However, the existing systematic reviews combined all exercise categories into a single analysis, which may have diluted the estimated effectiveness of the PA intervention.

Previous systematic reviews have examined the effectiveness of PA interventions for pregnant women both with normal weight, overweight or obesity. Some of these reviews identified that PA intervention significantly improved the health outcome in normal-weight pregnant women and infants, while some found that there is no significant different between experimental group and control group [21,22]. These studies did not differentiate PA interventions by type, intensity, or duration, which may result in difference effectiveness [23]. The intensity, type, and duration of exercise interventions significantly influence their effectiveness across various health outcomes for pregnant women. Supervised, moderate-intensity aerobic exercise has been recognized as an evidence-based approach for alleviating depressive symptoms [24]. This is clinically meaningful in maternal health, as accumulating evidence indicates that antenatal depressive symptoms are associated with an elevated risk of excessive gestational weight gain [25]. Extensive research has been conducted on PA interventions targeting types, intensities, lasting from 5 weeks to 30 months, for children and adolescence with overweight or obesity [26]. However, there are still very few studies specifically focusing on the intervention characteristics of PA type, intensity, and duration to reduce GWG in overweight or obese pregnant women [27]. These differences and lack of evidence emphasize the need for comprehensive, recent meta-analyses to assemble robust evidence. Conventional meta-analytic methods are constrained by evaluating the effectiveness of individual interventions because of restricted data accessibility. Yet, no extensive studies have thoroughly assessed and specific PA categories, duration, intensity for treating excessive GWG in overweight or obese pregnant women. Therefore, the aim of this study was to review the current commonly used PA intervention type, duration, and intensity; a systematic review and meta-analyses were used to assess the effect of PA treatments on GWG in pregnant women with overweight or obesity.

## 2. Materials and Methods

### 2.1. Protocol

Our study methodology conforms to the procedures set out in the Preferred Reporting Items for Systematic Reviews and Meta-Analyses (PRISMA and MA) and is consistent with the guidance of the Cochrane Collaboration. To promote transparency and reproducibility, we have included the PRISMA checklist in the Supplementary Materials (see Supplementary Table S1). In addition, we registered this meta-analysis with PROSPERO (registration number CRD420251069961) to ensure compliance with established standards for registering and reporting systematic reviews.

### 2.2. Search Strategy and Study Selection

Two distinct researchers examined four online databases (EBSCOhost, Embase, PubMed, and Web of Science) for pertinent studies published until August 2025. The search strategy was constructed following the PICO framework and specified: the target population comprises pregnant women classified as individuals with obesity or overweight. PA is the intervention being examined, which is evaluated in comparison with standard prenatal care. The primary outcome measured is GWG. The included studies are randomized controlled trails (RCTs).

A search strategy using keywords linked by Boolean operators was implemented to refine retrieval. Terms included: (“mother” OR “ maternal” OR “ pregnant” OR “ pregnancy women”) AND (“excess fat” OR “ obesity” OR “ excess weight” OR “ obese “ OR “overweight “) AND (“randomized controlled trial” OR “ RCT”) AND (“management” OR “ treatment” OR “ program” OR “intervention”). The Supplementary Materials contain the entire search query (Supplementary Table S2).

To maximize coverage, supplementary manual searches were also performed on relevant literature reviews, key abstracts from major international conferences, meta-analyses, and study citations. The initial search results were subjected to a structured screening workflow undertaken independently by two reviewers who were blinded to each other’s decisions to minimize bias. Screening and reference management were conducted with EndNote (version X9, Thompson ISI ResearchSoft, Carlsbad, CA, USA), and duplicate records were identified and removed. Thereafter, full-text eligibility was assessed independently by two experts; conflicts were settled by dialogue or, if required, by speaking with another expert.

Inclusion and exclusion criteria

The eligibility framework for this meta-analysis was determined according to predefined core parameters, namely the study comparator, outcomes, intervention, population, and study design. The precise requirements for admission were delineated as follows:

1. Pregnant women with overweight or obesity without additional diseases.
2. Physical activity as an intervention without limitation in categories, duration, and intensity, delivered either by a professional or through eHealth/mHealth platforms.
3. Comparison between intervention and control groups.
4. Availability of changed gestational weight gain outcome data.
5. Randomized controlled trials with parallel groups.

The study imposed no limitations on gestational stage, geographical region, ethnicity, language, or publication year. The categorization of human–computer interaction, eHealth, and mHealth interventions was based on their respective definitions.

Exclusion criteria included: 1. conference abstracts, study protocols, or non-RCT designs; 2. studies conducted in non-human populations; 3. interventions not related to PA; 4. unavailability of outcome data.

### 2.3. Data Acquisition and Management Strategy

Data extraction from the included RCTs was conducted independently by two reviewers, each blinded to the other’s outcomes. A structured extraction form, designed in accordance with established methodological standards, was employed to ensure consistency and minimize bias [28]. The extracted variables comprised final follow-up, outcomes for GWG evaluated at both baseline and gestational week, sample size, intervention characteristics, participants’ age (reported as median or mean), year of publication, and the name of the first author. If there was no consensus, an independent expert arbitrated disputes regarding data extraction.

### 2.4. Assessment of Study Quality

The methodological quality of the included RCTs was assessed using the Cochrane Collaboration’s Risk of Bias (ROB) tool, in line with established guidance [29]. The following seven domains were assessed independently by two reviewers following the guidelines of the Cochrane Handbook (version 5.1.0): (1) generating sequences at random; (2) hiding allocation; (3) ensuring that participants and staff are blinded; (4) ensuring that outcome assessments are blinded; (5) ensuring that outcome data is full; (6) selectively presenting results; and (7) addressing additional forms of bias. Based on these evaluations, each trial was categorized as presenting a “unclear”, “low”, or “high” risk of bias. The two reviewers made their initial judgments independently; any discordances were resolved through discussion or, when consensus was not achieved, by arbitration from an independent third reviewer. Risk-of-bias determinations were recorded and managed using RevMan software (version 5.4, Cochrane Collaboration).

### 2.5. Statistical Analysis

To determine effect sizes associated with continuous variables, we extracted group means and standard deviations (SD) for each study and presented the weighted mean difference (MD) for each estimate. Additionally, the aggregate effect magnitude was independently evaluated using 95% confidence intervals (CIs) and pooled MDs. Effect sizes were expressed as Cohen’s *d* to enable comparison of standardized mean differences across trials. The synthesis of results was first undertaken using a fixed-effect model. When evidence of between-study heterogeneity was observed, analyses were re-estimated under a random-effects framework to provide more conservative pooled estimates. Heterogeneity was quantified with the Q statistic and I^2^ index, with thresholds of *p* < 0.10 or I^2^ > 50% considered indicative of meaningful inconsistency. Plotting funnel plots allowed us to identify possible publishing bias. Statistical analysis of MA in this study was performed using Stata/SE 17.0 (Stata Corp).

## 3. Results

### 3.1. Search Findings and Study Profiles

**Figure 1:**
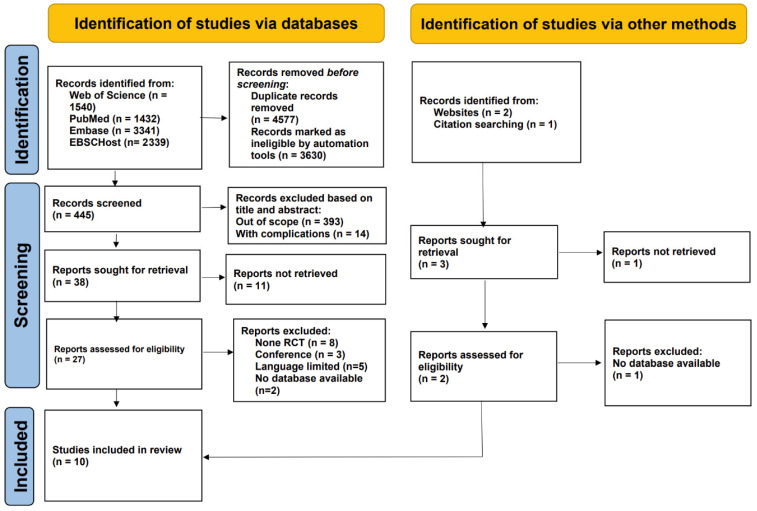
The PRISMA flow diagram illustrates the detailed process used for selecting articles included in this evaluation.

A total of 8652 records were originally discovered, supplemented by three more records obtained from bibliographies and global trials registries. Following the drop of 4577 duplicate records and 3630 entries deemed ineligible by automated tools, 407 articles were excluded after screening of titles and abstracts. Subsequently, qualifications were checked for 41 full-content publications. Among them, 31 were excluded for the following reasons: 12 full texts could not be retrieved, 8 did not employ an RCT design, 3 were conference abstracts, 5 were published in languages outside the inclusion criteria, and 3 were not available on accessible databases. Ultimately, 10 studies satisfied the inclusion criteria, enrolling pregnant women, with 622 participants in the intervention groups and 528 in the control groups (Figure 1). The participants’ ages ranged from 22 to 38 years. All included studies were RCTs, published between 2009 and 2018, in English, and investigated interventions spanning from early pregnancy through delivery (Table 1).

### 3.2. Intervention Characteristics

Table 2 presents the characteristics of the interventions from the eligible studies, encompassing details such as the delivery setting, behavior targets, modality and intensity, intervention duration, control group, and main outcome. The prenatal PA interventions duration varied in the reviewed studies, with the earliest beginning at 6 weeks of gestation [30] and the latest around the 20th week [38,39]. The articles included various types of PA, including walking (n = 1), aerobic exercise (n = 6), endurance training (n = 2), strength training (n = 3), and nutritional advising (n = 2). With respect to intervention intensity, five studies implemented moderate-intensity exercise protocols, one utilized low-intensity activity, and three adopted a combination of both low- and moderate-intensity exercises. In one study, the authors did not disclose the intensity of the intervention. The duration of PA sessions varied across studies, with six reporting sessions lasting between 30 and 60 min, one study implementing sessions shorter than 30 min, and another study not specifying the session length.

Intervention settings varied across studies, with four conducted in clinical environments, three in non-clinical community-based facilities, and one delivered at home through a mobile application. The specific delivery setting was not reported in two studies (Table 2). Among the included studies, interventions commenced during the first trimester in two cases and the second trimester in seven, while one study did not specify the initiation time. The duration of interventions ranged from 10 to 34 weeks. Control conditions across the included studies varied. Five studies provided participants in the control group with routine prenatal care. One study involved routine PA. Another trial offered educational sessions focusing on both PA and dietary guidance. In one study, the control group adhered to the hospital’s standard care protocol for obese pregnant women. Additionally, one trial included basic counseling on diet and PA, while another incorporated weekly relaxation and focus group discussions. The primary study outcomes were related to GWG, GDM, body mass index (BMI), and fetal birth outcomes.

### 3.3. Qualitative Summary of Included Studies

The included RCTs demonstrated notable diversity in PA modalities, session frequency, supervision levels, and delivery modes. Aerobic exercise (n = 6) was the most common modality, followed by combined aerobic–resistance programs (n = 3) and endurance-based activities (n = 2). Most interventions (n = 6) used moderate-intensity exercise for 15–60 min per session. Clinic-based supervised programs generally achieved higher adherence compared with home-based or mHealth formats. Regarding the control group settings, two studies incorporated basic dietary counseling or educational elements, and one study’s standard care also included guidelines for PA and D, PA remained the primary active component of the intervention across all experimental groups in the included trials.

### 3.4. Study Quality Assessment

**Figure 2:**
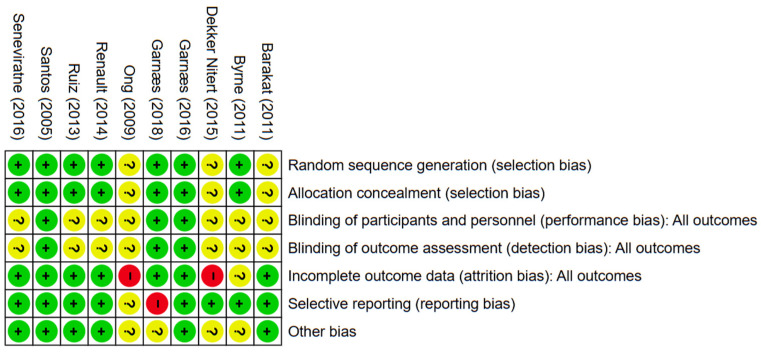
The evaluation of risk of bias. Green, red, and yellow indicate low, high, and unclear risk of bias, respectively; “+”, “−”, and “?” denote low, high, and unclear risk of bias [30,31,32,33,34,35,36,37,38,39].

**Figure 3:**
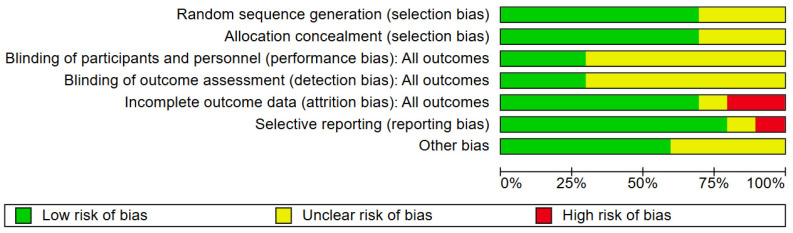
The risk of bias graph.

The outcomes of the quality evaluation showed that in the domains of random sequence generation and allocation concealment, 7 (70%) of 10 included papers were assessed to have a low risk of bias, while the remaining 3 (30%) included articles [30,32,35] lacked clear methodological detail and were rated as unclear. In the domain of blinding of participants and personnel, three (30%) were deemed low risk; seven (70%) included articles were unclear (Figure 2 and Figure 3). One (10%) trial [34] was judged to have a high risk of bias due to selective outcome reporting, eight (80%) trials demonstrated a low risk. The domains of performance and detection bias were consistently rated as unclear in seven (70%) studies, primarily due to inadequate information regarding whether outcome assessors were blinded to group allocation. Seven (70%) trials were assessed as having a low risk of bias concerning incomplete outcome data. In contrast, two (20%) were categorized as exhibiting a significant risk of bias. Six (60%) articles were categorized as low risk for other bias owing to inadequate sample size, four (40%) articles [31,32] were classified as unclear.

### 3.5. Heterogeneity Analysis

**Figure 4:**
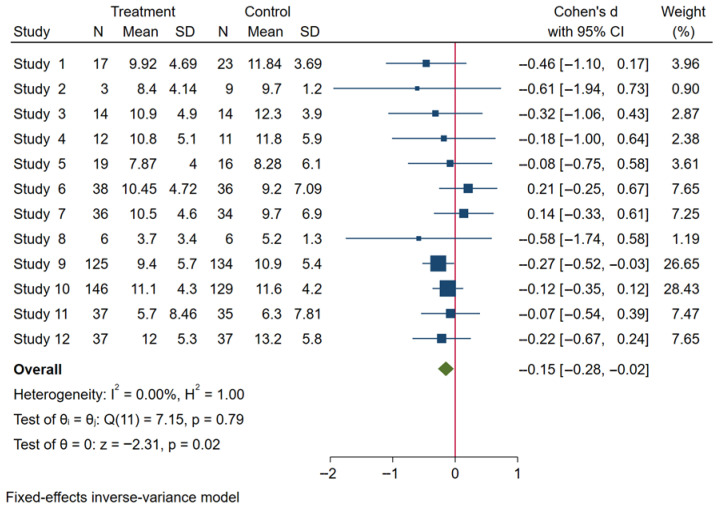
Forest plot analysis of exercise intervention effects on GWG in pregnant women.

**Figure 5:**
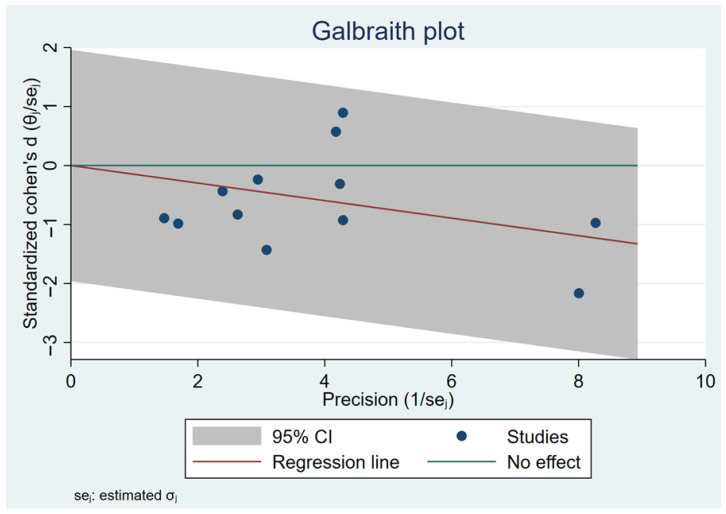
Galbraith plot assesses heterogeneity across 12 included studies.

**Figure 6:**
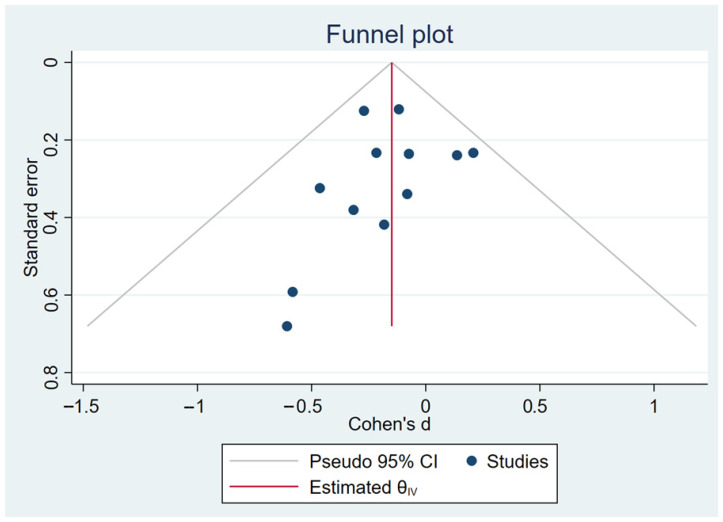
Funnel plot representation of publication bias.

The analysis was performed using a fixed-effects inverse-variance model. Heterogeneity analysis showed I^2^ = 0% (*p* = 0.79), indicating low between-study variability and high consistency among the included studies (Figure 4). The Galbraith plot in Figure 5 illustrates that all 12 arms fall within the 95% confidence interval boundaries, indicating good consistency across studies and no major sources of heterogeneity. Exercise intervention significant reduced in GWG amount pregnant women with OB or OW. The funnel plot in Figure 6 displays all 12 included trials, each represented by a blue dot. Ten of the twelve studies fall well within these limits, and the remaining two lie close to the boundaries, yielding an approximately symmetrical distribution about the vertical axis. This symmetry and the fact that all studies cluster within or near the funnel region indicate minimal small-study effects and no clear evidence of publication bias.

## 4. Discussion

For pregnant women with overweight or obesity, this meta-analysis is rare among systematic reviews as it synthesizes data from RCTs that look at how structured PA treatments affect GWG. Total of 12 RCTs involving 1107 participants were included. Our pooled analysis demonstrated that for moderate-intensity PA interventions, modest reductions were observed across a range of PA modalities. Furthermore, although three included studies utilized combined aerobic and resistance training, the available evidence does not allow firm conclusions regarding the superiority of any specific modality in GWG. Evidence indicates that limiting excessive GWG is associated with a lower risk of gestational diabetes mellitus, hypertensive disorders, and cesarean delivery [40]. Therefore, although the absolute difference observed in our meta-analysis was modest, it may still contribute to improved maternal–fetal outcomes at the population level. Additionally, the low heterogeneity observed (I^2^ = 0%) suggests consistent intervention effects across studies. Although statistical heterogeneity was low (I^2^ = 0%), substantial clinical heterogeneity existed across studies, including variability in exercise modality (aerobic, resistance, mixed), supervision level, intervention duration, delivery mode (clinic vs. home vs. mHealth), and comparator conditions. In addition, comparator conditions varied considerably across studies, ranging from routine prenatal care to structured diet counseling, PA education, or relaxation sessions. Such variability in control conditions may attenuate or inflate observed intervention effects and should be considered when interpreting pooled estimates. These differences may influence the generalizability of pooled estimates. These findings reinforce the growing recognition of PA as a critical, modifiable key contributor to maternal and fetal health outcomes. It is well known that excessive GWG increases the likelihood of adverse pregnancy outcomes, such as gestational diabetes mellitus, preeclampsia, and cesarean delivery. Guidelines from authoritative bodies such as the ACSM and the WHO emphasize the importance of structured PA in pregnancy, particularly for women with elevated pre-pregnancy BMI [15,16]. Since the American College of Obstetricians and Gynecologists (ACOG) issued more open recommendations on prenatal exercise in 2002, research in this domain has expanded significantly [41]. The included trials, spanning from 2005 to 2018, reflect this growth in scientific inquiry, with studies increasingly incorporating methodological advances in intervention delivery and outcome assessment.

However, the paucity of recent studies may be partly attributable to the disruption caused by the COVID-19 pandemic, shifting research priorities, and logistical challenges. Particularly, the geographical distribution of the included studies was heavily skewed toward high-income countries, especially Europe and Australia. In several Asian contexts, traditional beliefs and societal norms may restrict vigorous or unsupervised PA during pregnancy. Furthermore, local clinical guidelines and PA recommendations tailored to pregnant populations are either underdeveloped or poorly disseminated in these regions. Limited research infrastructure and competing public health priorities, such as infectious disease control, may further constrain opportunities for large-scale trials. The limited number of eligible studies identified in this review highlights the logistical, ethical, and methodological challenges inherent in conducting prenatal PA RCTs. Pregnancy presents unique constraints, including heightened safety concerns, risk aversion among potential participants, and complex physiological changes that complicate trial design and execution. Recruitment was particularly challenging, as illustrated by several included trials that enrolled fewer than 30 participants. Such limitations were flagged in our risk of bias assessment, particularly in relation to imprecision and incomplete outcome data.

Emerging technologies, such as mHealth platforms, represent promising avenues to overcome some of these barriers. One included study employed a mobile app-based PA intervention, reflecting a growing shift toward remote, scalable, and accessible exercise programs in prenatal care. Although mHealth interventions have been successfully implemented in other populations, including adolescents with obesity [42] and adults with overweight [43], their adoption in pregnancy has been more limited, potentially due to safety concerns and varying levels of digital literacy among expectant mothers. Nonetheless, the potential of mHealth tools, such as wearable devices, real-time feedback systems, and app-based coaching, to improve adherence and participant engagement is considerable [44,45,46]. Future research could systematically evaluate the comparative effectiveness of mHealth versus traditional supervised PA interventions in pregnant women. This is particularly true in populations with overweight and obesity, where tailored approaches may be critical to achieving clinically meaningful outcomes. Several methodological considerations emerged from this analysis. Wide variation in PA intervention characteristics, ranging from 10 to 34 weeks in duration, 3 to 5 sessions per week, and 15 to 60 min per session, likely contributed to inconsistent findings across individual trials. Furthermore, adherence to PA interventions during pregnancy is also shaped by psychosocial factors such as fatigue, anxiety about fetal safety, body-image concerns, lack of social support, and competing family responsibilities. Shorter or lower-intensity programs may have provided insufficient physiological stimulus to induce measurable changes in GWG, while longer, more intensive interventions may have encountered adherence challenges. However, few studies assessed intervention fidelity or monitored adherence systematically. Without standardized adherence reporting, it is difficult to determine whether null or small effects reflect insufficient intervention dosage rather than true inefficacy. This variability makes it imperative for future trials to adopt standardized protocols based on established guidelines such as those from ACOG and WHO, while closely monitoring adherence to ensure that the intervention dosage is adequate to elicit the desired health benefits [47,48,49].

Additionally, most interventions commenced during the second trimester, likely due to the alleviation of early gestational symptoms that often hinder exercise engagement in the first trimester [50]. This timing aligns with increased psychological and physical readiness among pregnant women to participate in structured exercise programs [51]. Future studies may investigate stage-specific exercise prescriptions that adjust intensity, duration, and modality to the unique physiological and psychological profiles of each trimester to optimize safety, adherence, and effectiveness. Risk of bias assessments revealed concerns related to selective outcome reporting, attrition bias, and incomplete blinding [50]. The challenges of implementing blinding in PA interventions were well-documented, particularly in studies requiring face-to-face supervision [52]. Furthermore, brief intervention sessions and the natural physiological changes accompanying pregnancy, such as increased body fat and fetal growth, may obscure detectable differences in maternal weight trajectories [53]. Notably, eight included trials did not systematically report maternal or fetal adverse events, limiting conclusions regarding the safety of different PA modalities. These factors call for more precise trial designs, longer intervention durations, and improved outcome measurement strategies to enhance future finding’s reliability and validity.

Among the limitations of the included studies is the exclusive focus on low- to moderate-intensity PA, reflecting current safety guidelines that limit high-intensity exercise during pregnancy [54]. However, emerging evidence suggests that well-supervised high-intensity interval training (HIIT) may offer additional cardiometabolic benefits, such as improved glycemic control, blood pressure regulation, and endothelial function in pregnant women with a normal weight [55]. This evidence raises the question of whether similar benefits could be safely extended to pregnant populations with overweight and obesity, who are at heightened metabolic risk. Future investigations may explore the feasibility, safety, and efficacy of HIIT protocols in this group under carefully monitored conditions. The findings will potentially expand the spectrum of safe and effective PA options.

### Strengths and Limitations

This meta-analysis presents several methodological advantages. It is the first to systematically differentiate PA interventions by type, intensity, and duration specifically for pregnant women with overweight and obesity. The study employed a comprehensive, multi-database search strategy and adhered rigorously to PRISMA guidelines, with dual independent review processes employed for study selection, data extraction, and quality assessment, thereby enhancing findings transparency and reproducibility. Low heterogeneity and stable results across sensitivity analyses further strengthen the reliability of the conclusions. By focusing on a high-risk population, this study contributes targeted, actionable evidence that can inform more personalized exercise recommendations in prenatal care. Nevertheless, several limitations of this meta-analysis should be acknowledged. The included RCTs were generally small in scale and showed substantial variability in intervention characteristics and methodological quality, which may reduce the precision and generalizability of the pooled estimates. Moreover, generalizability is further constrained by the geographical concentration of studies in high-income countries, where differences in cultural norms, healthcare access, and PA recommendations may limit the applicability of the findings to low- and middle-income populations. The exclusive reliance on English-language publications may have introduced language bias and excluded relevant studies from non-English-speaking regions. Additionally, despite efforts to include only participants with overweight or obesity, potential heterogeneity in baseline characteristics across studies may have introduced selection bias. Although funnel plot inspection suggested minimal publication bias, the small number of included trials limits the reliability of this assessment. Furthermore, several included trials used multi-component interventions in which PA was delivered together with dietary or behavioral guidance. These co-interventions may influence outcomes commonly attributed to PA; however, the available data did not allow separate analysis of individual components. Therefore, the independent effect of PA should be interpreted with caution. Pregnant women with overweight or obesity present unique physiological, psychological, and behavioral profiles that necessitate tailored intervention strategies. These strategies were not consistently addressed across all included trials.

Overall, the evidence supports the modest benefits of structured PA in controlling GWG in pregnant women with overweight and obese. Future studies should adopt standardized protocols, robust adherence monitoring, and diversified delivery models to strengthen the evidence base and improve intervention designs.

## 5. Conclusions

This meta-analysis found that PA interventions had a modest but statistically significant effect on reducing gestational weight gain among pregnant women with overweight and obesity. Moderate-intensity exercise is most frequently utilized intervention strategy for managing GWG. Differentiating the characteristics of interventions by type, intensity, and duration provided valuable insights into optimizing exercise prescriptions for this high-risk population. However, the RCTs included in this study generally had small sample sizes and methodological variability, which limited the strength and generalizability of the findings. Future high-quality RCTs with standardized designs, innovative delivery methods, such as digital health platforms, and long-term follow-up would be necessary to strengthen the evidence base and improve intervention strategies. Overall, these findings emphasize the importance of early, tailored PA interventions to support healthy pregnancies and reduce long-term health risks associated with maternal obesity.

## Figures and Tables

**Table 1:** The demographic features of included studies.

Author (Year)	Ethnicity/Country (%)	Age (M ± SD)	Gestation Week (M ± SD)	Sample Size (N)	Participant Type
Barakat (2011) [30]	Caucasian (100%)	I: 32 ± 4.0	≥6 weeks	I:40	OB/OW
		C: 31 ± 3.0		C:43	
Byrne (2011) [31]	Australia (100%)	I: 31.1 ± 3.0	≤12 weeks	I: 11	OB
		C: 31.6 ± 3.1		C: 12	
Dekker Nitert (2015) [32]	Australia (100%)	I: 33.3 ± 5.6	12 weeks	I: 16	OB
		C: 30.8 ± 4.9		C: 19	
Garnæs (2016) [33]	Norway (100%)	I: 31.3 ± 3.8	≥12 weeks	I: 38	OB/OW
		C: 31.4 ± 4.7		C: 36	
Garnæs (2018) [34]	Norway (100%)	I: 31.6 ± 3.6	16–18 weeks	I: 36	OB/OW
		C: 31.3 ± 4.6		C: 34	
Ong (2009) [35]	Australia (100%)	T: 30 ± 4.0	18 weeks	I: 6	OB
				C: 6	
Renault (2014) [36]	Caucasian (IA: 98%, IB: 98% and C: 97%)	IA: 31.2 ± 4.4	<16 weeks	IA: 130	OB
		IB: 30.9 ± 4.9		IB: 125	
		C: 31.3 ± 4.2		C: 134	
Ruiz (2013) [37]	Spain (100%)	I: 31.6 ± 4.0	5–6 weeks	I: 146	OB/OW
		C: 31.9 ± 4.0		C: 129	
Santos (2005) [38]	Brazil (100%)	I: 26.0 ± 3.4	I: 17.5 ± 3.3	I: 37	OW
		C: 28.6 ± 5.9	C: 18.4 ± 3.9	C: 35	
Seneviratne (2016) [39]	Pacific Islander (I: 29%, C: 29%), Maori (I: 13%, C: 14%), New Zealand European or other (I: 58%, C: 57%)	I: 31.6 ± 4.6	20 weeks	I: 37	OB/OW
		C: 31.1 ± 5.2		C: 37	

Note: I, intervention group; C, control group; OB, obesity; OW, overweight; M, mean; SD, standard deviation.

**Table 2:** The intervention protocol characteristics of included studies.

Author (Year)	Delivery Setting	Behavior Targets (Type)	Modality and Intensity	Intervention Duration	Control Group	Main Outcome
Barakat (2011) [30]	At non-clinic setting by exercise specialists	PA (light to moderate)	85 training sessions (35–45 min/session)	From 6 gestation weeks to term	Routine prenatal care	50 g MGS GDM Body weight BMI GWG
Byrne (2011) [31]	At clinic	PA (walking)	Monthly individual, phone call or group sessions (NR)	From 15 to 30 gestation weeks	Routine prenatal care	Height Skinfold thickness BMI GWG RMR Walking speed Metabolic cost of walking Body composition
Dekker Nitert (2015) [32]	Location was not reported	PA (individualized), E (PA and D guidance)	Monthly individual and group sessions (NR)	From 12 to 36 gestation weeks	E (PA and D guidance)	GWG PAL Fasting glucose
Garnæs (2016) [33]	At clinic	PA (moderate, endurance and resistance training)	3 times/week group sessions (60 min/session) and 1 times home exercise per week (50 min/sessions	From 12–18 to 34–37 gestation weeks	Routine prenatal care	GWG
Garnæs (2018) [34]	At clinic	PA (endurance training and resistance training, home exercise, pelvic floor muscle exercises)	PA (3 times/week, 35 min endurance training and resistance training; daily home exercise; daily pelvic floor muscle exercises)	From 12–18 gestation weeks until delivery	Routine prenatal care	GWG BMI GDM
Ong (2009) [35]	At non-clinic setting	PA (moderate, stationary cycling)	Home-based exercise (3 times/week, 50 min/session)	10 weeks	Routine prenatal care	PAL GWG OGTT
Renault (2014) [36]	At clinic	PA (11,000 steps daily), D (Mediterranean-style diet)	Text message, 11–13 phone calls (1 time per 2 weeks)	From 16 gestation weeks to term	Received the usual hospital standard regimen for obese pregnant women	GWG PAL
Ruiz (2013) [37]	NR	PA (light- to moderate-intensity aerobic and resistance exercises)	85 training sessions (3 times/week, 50–55 min/session).	From 9 to 38–39 gestation weeks	Simple dietary and PA counseling	GWG
Santos (2005) [38]	At non-clinic setting	PA (moderate aerobics)	Group exercise sessions (3 times/week, 1 h/session)	From 20 to 32 gestation weeks	Weekly relaxation and focus group discussions	VO2 GWG
Seneviratne (2016) [39]	Mobile application At home	PA (moderate stationery cycling)	67 home-based exercises (3 to 5 times/week, 15 to 30 min/session)	From 20 to 35 gestation weeks	Routine physical activity	Birth outcomes GWG

Note: MGS: maternal glucose screen; GDM: gestational diabetes mellitus; RMR: resting metabolic rating, PAL: physical activity level; OGTT: oral glucose tolerance test; VO2: the values of oxygen uptake at the anaerobic threshold; NR: no report; PA: physical activity; D: dietary; E: education; GWG: gestational weight gain.

## Data Availability

The original contributions presented in this study are included in the article/Supplementary Materials. Further inquiries can be directed to the corresponding authors.

## Supplementary Materials

The following supporting information can be downloaded at: https://www.mdpi.com/article/10.3390/healthcare13243319/s1, Table S1. PRISMA_Checklist [56]. Table S2: Search_Strategy.
