# Supplementary material for: A Systematic Review and Meta-Analysis of the Effects of Various Physical Activity Interventions in Pregnant Women with Overweight or Obesity
Source: Healthcare (Basel). 2025 Dec 18;13(24):3319. doi: 10.3390/healthcare13243319 (PMC12732830; doi:10.3390/healthcare13243319)
Supplement: Supplementary file 1 [file healthcare-13-03319-s001.zip › Supplementary_Table_S2_Search_Strategy.pdf]

Supplementary Table S2 Search strategy for **Medline (via PubMed), EBSCHost, Embase and Web of Science** Core Collection databases. Search strategy for **Medline (via PubMed)** (inception-15 August 2025).

**Category**	**Search terms**
Population	**#1** pregnan* OR pregnant wom?n OR antenatal OR prenatal OR “prenatal period” OR “expectant mothers” OR “prenatal care” [All Fields] 1,238,808<br>**#2** obesity OR overweight OR obes* OR adipos* OR “body mass index” OR bmi OR waist OR “body fat” OR “skin fold” OR skinfold [All Fields] 915,495<br>**#3** **#1** AND **#2** 54,291
AND<br>Intervention	**#4** exercise OR "physical activity" OR "physical exertion" OR swim* OR walk* OR danc* OR cycl* OR bicycl* OR hiking OR "tai ji" OR tai-ji OR "tai chi" OR yoga OR qigong OR "qi gong" OR sport* OR "physical training" OR "strength training" OR "weight training" OR "resistance training" OR "balance training" OR "aerobic training" OR "anaerobic training" OR "endurance training" OR "muscle training" OR exergame OR "active video game" OR wii OR kinect OR pilates OR feldenkrais OR "motor activity" OR "cardiac rehabilitation" [All Fields] 2,972,994
AND<br>Outcome	**#5** “gestational weight gain” OR “pregnancy weight gain” OR “maternal weight gain” OR “postpartum weight retention” OR “weight control” OR “weight change” OR “weight gain” OR weight OR “body mass” [All Fields] 2,172,834
AND<br>Study Design	**#6** random* OR rct OR “clinical trial” OR “intervention study” OR “interventional study” [All Fields] 2,300,967
Result	**#7 #3 AND #4 AND #5 AND #6** 1,432

Search strategy for **Embase** (inception-15 August 2025).

**Category**	**Search terms**
Population	**#1** 'pregnan*'/exp OR 'pregnant wom?n'/exp OR 'antenatal'/exp OR 'prenatal'/exp OR 'prenatal period'/exp OR 'expectant mothers'/exp OR 'prenatal care'/exp 1,378,244<br>**#2** 'obesity'/exp OR 'overweight'/exp OR 'obes*'/exp OR 'adipos*'/exp OR 'body mass index'/exp OR 'bmi'/exp OR 'waist'/exp OR 'body fat'/exp OR 'skin fold'/exp OR 'skinfold'/exp 1,521,190<br>**#3 #1** AND **#2** 91,066
AND Intervention	**#4** 'exercise'/exp OR 'physical activity'/exp OR 'physical exertion'/exp OR 'swim*'/exp OR 'gym*'/exp OR 'walk*'/exp OR 'danc*'/exp OR 'jog*'/exp OR 'run*'/exp OR 'cycl*'/exp OR 'bicycl*'/exp OR 'hiking'/exp OR 'tai ji'/exp OR 'tai-ji'/exp OR 'tai chi'/exp OR 'yoga'/exp OR 'qigong'/exp OR 'qi gong'/exp OR 'sport*'/exp OR 'physical training'/exp OR 'strength training'/exp OR 'weight training'/exp OR 'resistance training'/exp OR 'balance training'/exp OR 'aerobic training'/exp OR 'anaerobic training'/exp OR 'endurance training'/exp OR 'muscle training'/exp OR 'exergame'/exp OR 'active video game'/exp OR 'wii'/exp OR 'kinect'/exp OR 'pilates'/exp OR 'feldenkrais'/exp OR 'motor activity'/exp OR 'cardiac rehabilitation'/exp 5,374,448
AND Outcome	**#5** 'gestational weight gain'/exp OR 'pregnancy weight gain'/exp OR 'maternal weight gain'/exp OR 'postpartum weight retention'/exp OR 'weight control'/exp OR 'weight change'/exp OR 'weight gain'/exp OR 'weight'/exp OR 'body mass'/exp 1,818,397
AND Study Design	**#6** 'random*'/exp OR 'RCT'/exp OR 'clinical trial*'/exp OR 'intervention study'/exp OR 'interventional study'/exp 3,857,224
Result	**#7 #3 AND #4 AND #5 AND #6** 3,341

Search strategy for **EBSCHost** (inception-15 August 2025).

**Category**	**Search terms**
Population	**#1** TX pregnan* OR pregnant wom?n OR antenatal OR prenatal OR prenatal period OR expectant mothers OR prenatal care 1,931,037<br>**#2** TX obesity OR overweight OR obes* OR adipos* OR "body mass index" OR bmi OR waist OR body fat OR skin fold OR skinfold 2,040,124<br>**#3** **#1** AND **#2** 100,476
AND<br>Intervention	**#4** TX exercise OR "physical activity" OR "physical exertion" OR swim* OR gym* OR walk* OR danc* OR jog* OR run* OR cycl* OR bicycl* OR hiking OR "tai ji" OR tai-ji OR "tai chi" OR yoga OR qigong OR "qi gong" OR sport* OR "physical training" OR "strength training" OR "weight training" OR "resistance training" OR "balance training" OR "aerobic training" OR "anaerobic training" OR "endurance training" OR "muscle training" OR exergame OR "active video game" OR wii OR kinect OR pilates OR feldenkrais OR "motor activity" OR "cardiac rehabilitation" 30,333,076
AND<br>Outcome	**#5** TX "gestational weight gain" OR "pregnancy weight gain" OR "maternal weight gain" OR "postpartum weight retention" OR "weight control" OR "weight change" OR "weight gain" OR weight OR "body mass" 4,287,251
AND<br>Study Design	**#6** TX random* OR RCT OR "clinical trial" OR "intervention study" OR "interventional study" 4,994,953
Result	**#7 #3 AND #4 AND #5 AND #6** 2,339

Search strategy for **Web of Science Core Collection databases** (inception-15 August 2025).

**Category**	**Search terms**
Population	**#1** ALL= (Pregnan* OR pregnant wom?n OR antenatal OR prenatal OR prenatal period OR expectant mothers OR prenatal care) 823,008
	**#2** ALL= (obesity OR Overweight OR obes* OR adipos* OR "body mass index" OR bmi OR waist OR body fat OR skin fold OR skinfold) 1,115,694
	**#3** **#1** AND **#2** 53,843
AND Intervention	**#4** ALL= (exercise OR "physical activity" OR "physical exertion" OR swim* OR gym* OR walk* OR danc* OR jog* OR run* OR cycl* OR bicycl* OR hiking OR "tai ji" OR tai-ji OR "tai chi" OR yoga OR qigong OR "qi gong" OR sport* OR "physical training" OR "strength training" OR "weight training" OR "resistance training" OR "balance training" OR "aerobic training" OR "anaerobic training" OR "endurance training" OR "muscle training" OR exergame OR "active video game" OR wii OR kinect OR pilates OR feldenkrais OR "motor activity" OR "cardiac rehabilitation") 7,319,458
AND Outcome	**#5** ALL=("Gestational weight gain" OR "Pregnancy Weight Gain" OR "Maternal Weight Gain" OR "Postpartum Weight Retention" OR "weight control" OR "weight change" OR "weight gain" OR weight OR "body mass") 2,752,578
AND Study Design	**#6** ALL= (random* OR RCT OR "clinical trial" OR "intervention study" OR "interventional study") 2,789,748
Result	**#7 #3 AND #4 AND #5 AND #6** 1,540
